# The Application of AI to Ecological Momentary Assessment Data in Suicide Research: Systematic Review

**DOI:** 10.2196/63192

**Published:** 2025-04-17

**Authors:** Ruth Melia, Katherine Musacchio Schafer, Megan L Rogers, Emma Wilson-Lemoine, Thomas Ellis Joiner

**Affiliations:** 1 Health Research Institute University of Limerick Limerick Ireland; 2 Psychology Department Florida State University Tallahassee, FL United States; 3 Department of Biomedical Informatics Vanderbilt University Medical Centre Nashville, TN United States; 4 Department of Psychology Texas State University San Marcos, TX United States; 5 Department of Psychological Medicine Kings College London London United Kingdom; 6 Department of Psychology University of Virginia Austin, TX United States

**Keywords:** ecological momentary assessment, artificial intelligence, machine learning, suicidal thoughts and behaviors, mobile health, mHealth

## Abstract

**Background:**

Ecological momentary assessment (EMA) captures dynamic processes suitable to the study of suicidal ideation and behaviors. Artificial intelligence (AI) has increasingly been applied to EMA data in the study of suicidal processes.

**Objective:**

This review aims to (1) synthesize empirical research applying AI strategies to EMA data in the study of suicidal ideation and behaviors; (2) identify methodologies and data collection procedures used, suicide outcomes studied, AI applied, and results reported; and (3) develop a standardized reporting framework for researchers applying AI to EMA data in the future.

**Methods:**

PsycINFO, PubMed, Scopus, and Embase were searched for published articles applying AI to EMA data in the investigation of suicide outcomes. The PRISMA (Preferred Reporting Items for Systematic Reviews and Meta-Analyses) guidelines were used to identify studies while minimizing bias. Quality appraisal was performed using CREMAS (adapted STROBE [Strengthening the Reporting of Observational Studies in Epidemiology] Checklist for Reporting Ecological Momentary Assessment Studies).

**Results:**

In total, 1201 records were identified across databases. After a full-text review, 12 (1%) articles, comprising 4398 participants, were included. In the application of AI to EMA data to predict suicidal ideation, studies reported mean area under the curve (0.74-0.86), sensitivity (0.64-0.81), specificity (0.73-0.86), and positive predictive values (0.72-0.77). Studies met between 4 and 13 of the 16 recommended CREMAS reporting standards, with an average of 7 items met across studies. Studies performed poorly in reporting EMA training procedures and treatment of missing data.

**Conclusions:**

Findings indicate the promise of AI applied to self-report EMA in the prediction of near-term suicidal ideation. The application of AI to EMA data within suicide research is a burgeoning area hampered by variations in data collection and reporting procedures. The development of an adapted reporting framework by the research team aims to address this.

**Trial Registration:**

Open Science Framework (OSF); https://doi.org/10.17605/OSF.IO/NZWUJ and PROSPERO CRD42023440218; https://www.crd.york.ac.uk/PROSPERO/view/CRD42023440218

## Introduction

### Suicide Research

Over 700,000 people die by suicide each year globally [1]. Despite considerable research examining suicide risk factors, suicidal behavior remains difficult to predict. While the link between suicide and mental health difficulties (such as depression and alcohol use disorders) is well established in high-income countries, many suicides occur in moments of crisis with a breakdown in the ability to cope with difficulties, such as relationship breakups or chronic pain and illnesses. Experiencing conflict, disaster, violence, abuse or loss, and a sense of isolation are strongly associated with suicidal behavior [1]. Suicide rates are also high among vulnerable groups who experience discrimination, such as refugees and migrants; Indigenous peoples; lesbian, gay, bisexual, transgender, and intersex persons; and prisoners [1]. A meta-analysis of 50 years of research called for a shift in focus from risk factors (eg, previous suicide attempt and substance use) to machine learning (ML)–based risk algorithms [2]. Theoretical advances in the suicide literature propose dynamic relationships between key factors that warrant innovative research methodologies for adequate investigations [3-6]. In parallel, real-time assessment of suicidal thoughts and behaviors and related variables, using ecological momentary assessment (EMA) [7,8], offers a means of providing moment-to-moment assessment, more suitable for investigating the complex interplay between psychological, social, biological, and cultural factors put forward by prominent theories [3,9,10].

### Ecological Momentary Assessment

EMA uses systematic and frequent assessments to gather momentary data regarding an individual’s thoughts, emotions, behaviors, physical symptoms, and contexts as they occur in real time and outside clinical and other controlled settings. Common categories of EMA include (1) diaries, (2) experience sampling, (3) event-based sampling [11], and (4) passive sensing through wearable devices [7]. Diaries typically assess experience in fixed intervals (often daily) and involve retrospective recall; experience sampling typically uses a device to signal to the respondent to report on an experience at random times throughout the day. Event-based sampling elicits reports at the time of a particular event. Passive EMA involves measuring physiological states such as sleep or physical activity, typically (but not necessarily) via electronic wearable devices [7].

Such assessments are often delivered using handheld devices (such as smartphones) capable of time-stamping responses to short self-report questions in daily life. These measurements provide many methodological advantages over conventional assessment strategies when studying variable phenomena, investigating intraindividual changes, or complex and dynamic relationships between variables [7]. In addition, recall biases encountered in conventional retrospective survey methods can be diminished by the momentary evaluation of experiences or behaviors. Ultimately, such timely assessments could inform immediate and individually tailored interventions, offering advantages such as accessibility, availability, and versatility [12].

EMA designs have been found to be feasible and acceptable when delivered online and to anonymous participants [13] and have yielded good predictive validity in the measurement of suicide-specific outcomes when compared to standardized measures [14]. Indeed, more individuals reported suicidal ideation through EMA than traditional (retrospective) self-report measures. A recent systematic review reported that EMA of suicidal thoughts and behaviors was acceptable for participants and did not increase risk [14]. Available models suggest that through the process of active reflection and self-monitoring, social desirability, or feedback processes, the intensity of symptoms may decrease due to the unusual attention on target symptoms. Frequent self-monitoring may be a generic active ingredient across a variety of evidence-based psychological interventions (eg, diary cards in dialectical behavior therapy). However, researchers have noted that EMA components are inconsistently reported [14]. On the basis of the review by Kivelä et al [14], it is advisable to prioritize more frequent but brief assessments over short time periods to establish higher compliance. Future research should aim to more systematically examine how increasing the number of daily prompts affects compliance rates in order to establish optimal sampling schedules that balance temporal coverage with participant burden. Gee et al [15] asserted that further research should use longitudinal study designs, harmonize datasets, and use ML techniques to identify patterns of proximal risk factors for suicide behaviors.

Using data from smartphones and wearable devices, incorporating active data (active input from the user) or passive data (input from sensors), to better understand human behavior and personalize treatment plans is often described as digital phenotyping. This moment-by-moment quantification of individual-level human phenotype in situ, using data from personal digital devices [16], is being explored in relation to addiction, borderline personality disorder (BPD), posttraumatic stress disorder, and, more recently, suicidality [15-19].

### Artificial Intelligence and ML

Reviews of artificial intelligence (AI) in suicide research completed by Bernert et al [20] and later by Lejeune et al [21] highlight the burgeoning research in this area and demonstrate the potential that AI holds for identifying individuals at risk of suicide. AI can be used to identify patterns in large datasets, generate risk algorithms, and determine the effects of risk and protective factors on suicide.

As a form of AI, ML strategies enable computer learning of advanced classifiers to improve the accuracy of suicide prediction using large datasets. Burke et al [22] identified three main goals of ML studies of suicide: (1) improve the accuracy of risk prediction, (2) identify important predictors and the interactions between them, and (3) model subgroups of patients. Studies focused on improving suicide risk prediction suggest the high predictive potential for this technology [23]. Researchers have used supervised learning models [24], including ensemble learning methods (eg, random forests [RFs]), naïve Bayes classification, logistic and least squares regression, decision trees (DTs), and support vector machines, as well as unsupervised learning models [25], such as neural networks, clustering algorithms, self-organizing maps, principal component analysis, and DTs, to investigate large suicide outcome datasets. Reviews of the ML and suicide prevention literature indicate that methods vary substantially across studies and range significantly in rigor and model testing, with retrospective studies more represented than prospective studies and supervised learning models generally used more frequently than unsupervised learning techniques [20]. Overall, AI and ML have emerged as promising ways to improve the detection of suicide risk [20,21,26].

Recent years have seen an increase in the use of EMA data collection methods in suicide research, often described in quantitative case series studies [27], as part of randomized controlled trials of mobile apps [28], and in the investigation of suicide theories [29]. Therefore, an assessment of current research using these methods with such data are timely. In addition, previous research showed wide variability in the design and reporting of EMA studies assessing mental health and called for unifying standards for EMA reporting in mental health research going forward [30]. An emerging body of EMA datasets involving suicide outcomes and related factors holds the potential to substantially improve our understanding of complex suicide processes. Leveraging such unique datasets by applying AI strategies may offer a pivotal opportunity to advance our theoretical understanding and enhance prediction. Prediction can only support suicide prevention if it informs scalable, accessible, and evidence-based interventions.

### Just-in-Time Adaptive Interventions

EMA and digital phenotyping approaches have evolved and informed the development of ecological momentary interventions (EMIs), whereby treatments “are provided to people during their everyday lives (ie, in real time) and in natural settings” [31]. Research evidence on the effectiveness of EMI in the context of suicide prevention is scarce despite advances in the technology used to operationalize it. In a review of EMIs in suicide prevention research, Jiménez-Muñoz et al [12] reported that many of the available interventions had not yet been clinically tested, but those that had been tested (10 studies) showed good rates of effectiveness and feasibility. The most widely used intervention model in EMI studies in suicide research is the safety plan, which consists of strategies to help individuals during a suicidal crisis.

Relatedly, Coppersmith et al [32] argued that just-in-time adaptive interventions (JITAIs) hold the potential for reducing suicide rates. They are described as “an intervention design that adapts the provision of support (eg, the type, timing, and intensity) over time to an individual’s changing status and contexts, with the goal to deliver support at the moment and in the context that the person needs it most and is most likely to be receptive” [33]. Just-in-time adaptive interventions are used, particularly in health behavior change research, to address the states of vulnerability or periods of heightened susceptibility to negative health outcomes, such as smoking, unhealthy eating, and heavy drinking [34,35]. The emergence of a vulnerable state is seen as a dynamic process in which stable influences (eg, marital status and gender) and transient influences (eg, suicidal ideation and access to means) interact. They aim to capitalize on states of opportunity, namely periods of heightened susceptibility to positive health behavior changes (eg, healthy eating and physical activity), and have been put forward as feasible where states of vulnerability or opportunity emerge rapidly, unexpectedly, and ecologically (ie, in the individual’s natural environment). Just-in-time adaptive interventions involve 4 key features: decision points, intervention options, tailoring variables, and decision rules. Bryan et al [36] argued that further research is needed before such interventions can be constructed and applied as suicide prevention interventions, for example, knowing at an individual level *when* an intervention would be most effectively delivered (decision points), *what* that intervention should be (intervention options), the source and amount of the intervention to be delivered and in what context (tailoring variables), and what decision rules will inform when to offer which intervention. Therefore, further investigation of the application of AI to EMA datasets examining suicide outcomes is a necessary step to inform this next generation of digital interventions in suicide prevention. To our knowledge, this is the first systematic review that aims to synthesize empirical research on the application of AI to EMA data in the study of suicidal thoughts and behaviors.

The objectives of this review are to (1) synthesize published research on the application of AI strategies to EMA data in the study of suicidal ideation and behaviors; (2) identify the methodologies, study type and quality, EMA data collection procedures, suicide outcomes studied, types of AI strategies applied, and results reported; and (3) develop an adapted framework for reporting AI applied to EMA data in mental health research.

## Methods

### Protocol and Registration

This review followed the recommendations of the PRISMA (Preferred Reporting Items for Systematic Reviews and Meta‐Analyses) guidelines [37]. The protocol has been preregistered on both PROSPERO (CRD42023440218) [38] and the Open Science Framework [39]. An account of changes to study protocol from preregistration is provided in Multimedia Appendix 1.

### Inclusion and Exclusion Criteria

Studies were required to meet the following inclusion criteria, which are summarized in Textbox 1: (1) original, empirical research published in a peer-reviewed journal; (2) evaluated specific suicide outcomes—suicidal ideation, self-harm, nonsuicidal self-injurious behavior, suicide attempt, death by suicide; (3) used EMA to investigate suicide outcomes or related risk factors; (4) applied AI to EMA data; and (5) written in English. Interventional studies, observational studies, case series, and case reports were all included because such methodologies are suited to longitudinal EMA data collection. “Gray literature” and conference abstracts were also searched. Qualitative studies, protocols, opinion pieces, unpublished theses, and editorials were not included. Specifically, the types of EMA data collection included were (1) diaries, (2) experience sampling, (3) event-based sampling, and (4) passive sensing.

**Study inclusion and exclusion criteria.**

**Inclusion criteria**

- Article type
  - Original empirical research published in a peer-reviewed journal
  - “Gray literature” and conference abstracts
- Language: written in English
- Methodology
  - Interventional studies, observational studies, case series, and case reports
  - Evaluated specific suicide outcomes—suicidal ideation, self-harm, nonsuicidal self-injurious behavior, suicide attempt, and death by suicide
  - Used ecological momentary assessment (EMA) to investigate suicide outcomes and/or related risk factors
  - Types of EMA data collection included diaries, experience sampling, event-based sampling, and passive sensing
  - Applied artificial intelligence to EMA data

**Exclusion criteria**

- Article type: protocols, opinion pieces, and unpublished theses and editorials
- Language: not written in English
- Methodology
  - Qualitative studies
  - Did not report on specific suicide outcomes—suicidal ideation, self-harm, nonsuicidal self-injurious behavior, suicide attempt, and suicide death
  - Did not use EMA as a data collection procedure
  - Did not use the types of EMA, namely diaries, experience sampling, event-based sampling, and passive sensing
  - Did not apply artificial intelligence to EMA data

### Search Strategy

The following databases were searched for published studies: PsycINFO, PubMed, Scopus, and Embase. Search dates were as follows: initial search—July 20, 2023 (inception to July 2023); additional search—August 15, 2023 (inception to August 2023); final search before submission—June 2, 2024 (inception to June 2, 2024). Keywords were based on the following 3 fields: suicide, AI, and EMA. A search strategy, adapted for each database, was built using the Boolean operators “AND” and “OR” and applied to titles and abstracts. Three groups of terms were combined: the first relevant to suicide outcomes, the second relevant to EMA, and the third relevant to the application of AI or ML strategies. For example, search terms on Embase were as follows: suicide (suicide* / suicide ideation / suicidal / suicide attempt / suicide death) AND AI (Artificial Intelligence / AI / Machine Learning / ML) AND EMA (EMA / Ecological Momentary Assessment / Experience Sampling / Ambulatory Assessment).

To limit potential selection bias, we did not apply any restriction in terms of geographical location or population, but this was screened and extracted by researchers following the initial search. Studies not written in English were excluded. Details of the search strategy are provided in Multimedia Appendix 2.

### Selection of Studies

Two researchers (RM and KMS) independently reviewed 10% (13/127) of the titles and abstracts, and 90% agreement was reached before the remaining 90% (114/127) of titles and abstracts were screened based on the inclusion criteria. The same process was repeated when reviewing the articles at the full-text stage. A third researcher was requested to make the final decision if consensus was not achieved. Citations were imported into EndNote (Clarivate), and duplicates were removed before being uploaded onto Rayyan (Rayyan Systems Inc) [40]. In addition, a manual search of reference lists from relevant published systematic reviews was conducted. Web of Science was used to undertake forward and backward citation searching of reference lists from included studies. On the basis of PROSPERO good practice guidance, searches were rerun just before the final analyses, and any further studies were identified and retrieved for inclusion. As high agreement was reached in the first screening (>90%), RM independently conducted the updated search and consulted with a second author (KMS) for any articles where there was uncertainty.

### Data Extraction and Management

Data were extracted using a predefined form, and the data extraction process followed the procedures reported by published reviews conducted in this area [20,21]. Specifically, data were extracted independently by one rater, and 30% of the data were double checked by another rater; as a high rate of agreement was reached, the first author extracted the remaining 70% of the data. The authors were not blinded to information such as study author, institution, or journal. Authors were contacted if key information could not be ascertained from the article, its supporting information materials, or a previous publication referenced in the article that listed more details of the sample characteristics and study procedure. Characteristics of included studies were summarized and included the information presented in Textbox 2, wherever available.

**Description of data extracted from included studies.**

- Study description (study author, year, country, journal, and study type)
- Participant characteristics (sample size, mean or median age and SDs, population type, and setting [eg, community and hospital]
- Ecological momentary assessment (EMA) study type (eg, observational, interventional, and both)
- EMA delivery mode or device (eg, mobile phone, website or online, and pen-and-paper mode)
- EMA method (eg, signal contingent, event contingent, and multiple)
- EMA characteristics (eg, total study duration in days), prompting frequency (eg, hourly, daily, and weekly), and incentive schedule (eg, flat rate and payment per EMA)
- Adherence to EMA (eg, average percentage of EMAs completed out of available prompts)
- Suicide outcomes reported (suicidal ideation, self-harm, nonsuicidal self-injury, suicide attempt, and suicide death) and how they were measured (eg, EMA method, measurement frequency, existing standardized measure or bespoke, and whether a single item or multiple items were used)
- Mental health and suicide-related outcomes assessed (eg, measures of low mood, anxiety, sleep, and physical activity) and how they were assessed (eg, standardized measures, EMA method, and measurement frequency)
- Monitoring and follow-up period used
- Type of artificial intelligence or machine learning strategy applied (eg, classification and regression tree and recurrent neural network)
- Results reported (eg, model accuracy, sensitivity, specificity, or root mean squared error for recurrent neural networks)

### Data Synthesis

Study outcomes are presented in a narrative synthesis. We descriptively reported on the study characteristics, study type, EMA data collection procedures used, study context (country and setting), study sample, device used, AI applied, and results reported. As a meta-analysis was not possible due to the small number of studies eligible for inclusion and the heterogeneity of outcomes reported, we presented the results reported in each published report. Where sufficient data were available, we presented an overview of the results reported by providing ranges and means. Study limitations were described narratively in published studies and were included in data extraction. Finally, new categories, in addition to those included in the CREMAS (adapted STROBE [Strengthening the Reporting of Observational Studies in Epidemiology] Checklist for Reporting Ecological Momentary Assessment Studies), were added, following discussion, to inform the development of a reporting framework.

### Quality Appraisal

Quality appraisal was conducted using CREMAS [41]. CREMAS identifies 16 items recommended to report on in EMA studies, which raters used for quality appraisal. A description of each item is available in Multimedia Appendix 3. The reporting checklist includes methodological features, such as EMA prompt strategy, monitoring periods, response latency, prompt frequency per day and interval, the type of technology used and its administration, alongside study design features to address potential sources of bias. The quality indicators were coded by one rater, with 30% double checked by a second rater. Discrepancies were resolved through discussion with a third author. As a high level of agreement was observed across raters, the first author completed coding on the remaining 70% of the quality indicator. As each criterion referred to a different aspect of study quality, the extent to which each study reported each of the 16 items is presented in the Results section.

### Ethical Considerations

This study did not require ethics approval as it summarized data from previously published studies.

## Results

### Study Selection

**Figure 1:**
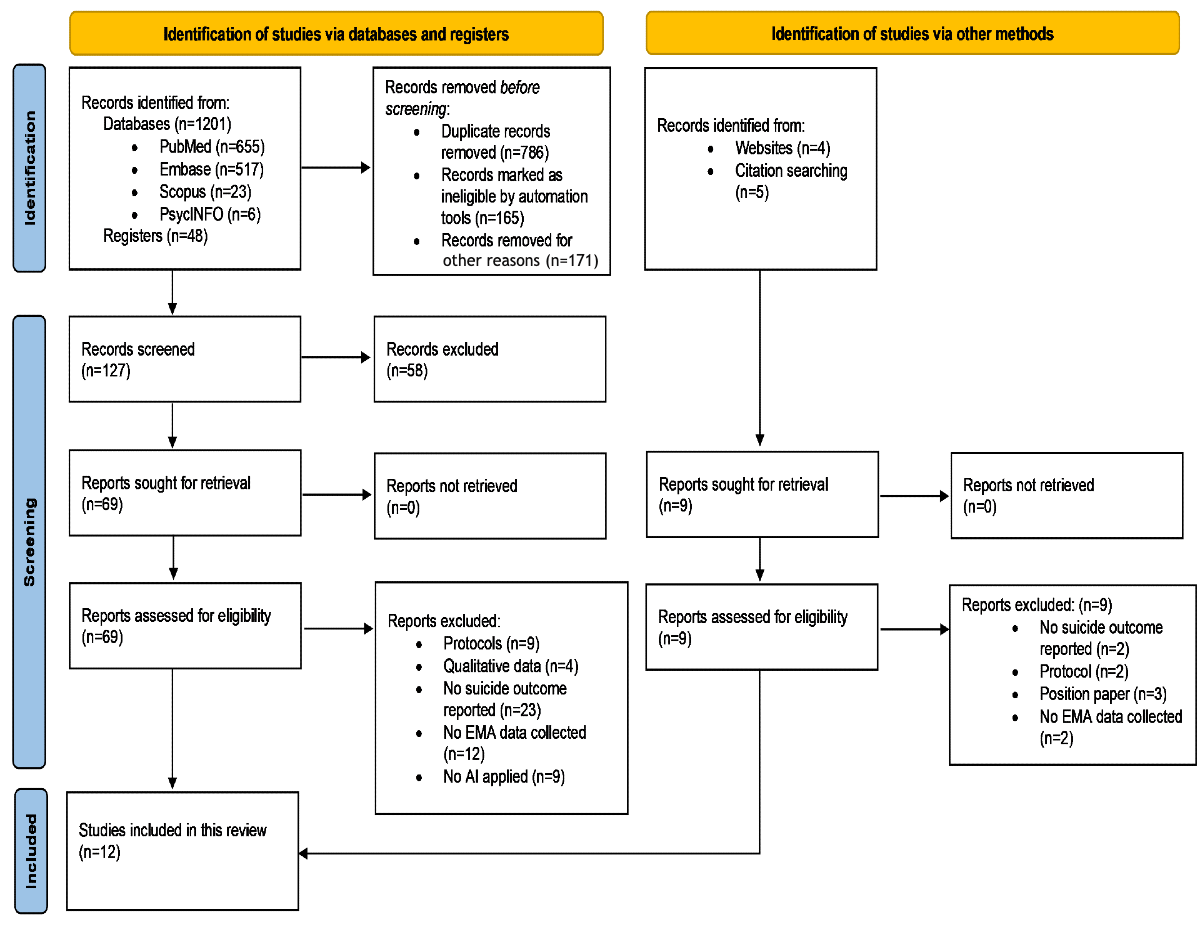
PRISMA flowchart. AI: artificial intelligence; EMA: ecological momentary assessment.

A total of 1201 records were identified across the following databases: PubMed (n=655, 54.54%), Embase (n=517, 43.05%), Scopus (n=23, 1.92%), and PsycINFO (n=6, 0.5%). Additional records were identified through registers (n=48). Of the total 1249 records identified, duplicates were removed (n=786, 62.93%), records were marked as ineligible by automation tools (n=165, 13.21%), and some were removed for other reasons (n=171, 13.69%), such as not being written in English. The remaining 127 records were screened (title and abstract), and 58 (45.7%) of these were excluded. In total, 69 full-text records were retrieved for review and assessment of eligibility. A PRISMA flowchart (Figure 1) was created to graphically depict the inclusion and exclusion of studies in the final review, together with a PRISMA checklist (Multimedia Appendix 4). Of the 69 full-text records retrieved, 58 (84%) were excluded. The 58 articles were excluded for the following reasons: report described a study protocol (n=9, 16%), reported only qualitative data (n=4, 7%), reported no suicide outcome data (n=23, 40%), reported no EMA data collection (n=13, 22%), and the application of AI was not reported (n=9, 16%).

In parallel, additional 9 records were identified through websites (n=4, 44%) and citation searches (n=5, 56%), but none were included in the review following screening and eligibility assessment. A total of 12 studies were included in this review, indicating that this is a small but rapidly growing area of research. A descriptive overview of included studies is provided in Table 1.

**Table 1:** Descriptive overview of the included studies. The overview includes the study authors, year of publication, countries where the studies were conducted, participant group, suicide outcomes studied, artificial intelligence (AI) strategy applied to the data, and device used for data collection.

Study	Location	Setting	Study population	Suicide outcome	AI strategy applied	Device used
Lei et al [42], 2023	China	Online	Gender and sexual minority individuals	Suicide ideation, self-harm, and suicide attempt	Light Gradient-Boosting Machine: the light gradient-boosting machine algorithm based on the GBDT^a^ algorithm	Not reported
Czyz et al [43], 2023	United States	Psychiatric hospital	Adolescent patients with psychiatric disorders (aged between 13 and 17 y)	Suicide ideation	Multilevel CART^b^	Smartphone
Horwitz et al [44], 2023	United States	Medical residency programs	Medical interns (first year)	Suicide ideation	Linear (elastic net regression) and nonlinear (random forest) algorithms	Wearable: Fitbit
Choo et al [45], 2022	United States	Psychiatric hospital	Adult psychiatry patients with BPD^c^	Suicide ideation	RNNs^d^	Not reported
Cobo et al [46], 2021	Spain	Psychiatric outpatient clinic	Adult psychiatry patients	Suicide ideation	Indian buffet process, a nonparametric Bayesian method	Smartphone
Czyz et al [47], 2023	United States	Emergency department	Young adult patients (aged between 18 and 25 y)	Suicide ideation	Mixed effects CARTs	Smartphone+wearable (Fitbit)
Kaurin et al [48], 2022	United States	Psychiatric outpatient clinic	Adult psychiatry patients with BPD	Suicide ideation	MSEM^e^	Smartphone
Bonilla-Escribano et al [49], 2023	Spain and France	Emergency and outpatient psychiatry clinic	Emergency and outpatient psychiatry patients	Suicide ideation and suicide behaviors	A GMM^f^ random forest algorithm	Smartphone
Marti-Puig et al [50], 2022	Spain	University mental health clinic and psychiatry clinic	University students, patients with BPD, and healthy controls	Self-harm	CART LOSO^g^ cross-validation	Smartphone
Peis et al [51], 2019	Spain	Psychiatric hospital	Adult psychiatry patients	Suicide ideation	RNNs	Smartphone
Choo et al [52], 2019	United States	Psychiatric hospital	Adult psychiatry patients with BPD	Suicide ideation	Compared MEMs^h^ with RNNs	Not reported
Wang et al [53], 2021	United States	Psychiatric hospital	Adult psychiatry inpatients	Suicide ideation and suicide attempt	Elastic net models	Smartphone

^a^GBDT: gradient boosting decision tree.

^b^CART: classification and regression tree.

^c^BPD: borderline personality disorder.

^d^RNN: recurrent neural network.

^e^MSEM: multiple structural equation modeling.

^f^GMM: Gaussian mixture model.

^g^LOSO: leave one subject out.

^h^MEM: mixed effects regression model.

### Quality of Included Studies

Overall, a high level of heterogeneity was observed across the included studies in terms of meeting the reporting standards (Table 2). Studies met between 4 and 13 of the recommended 16 items, with an average of 7 items met across studies. Studies performed poorly in their reporting of item 3 (“description of EMA training procedures for participants”) and item 14 (“report on the treatment of missing data”). Studies tended to consistently meet item 5 (“state the number of waves in the study”), item 7 (“indicate the EMA prompting strategy used”), and item 8 (“state the intended frequency of prompts per day”).

**Table 2:** Quality appraisal of the included studies using the CREMAS (adapted STROBE [Strengthening the Reporting of Observational Studies in Epidemiology] Checklist for Reporting Ecological Momentary Assessments Studies) [41], presence or absence of each of the 16 CREMAS items, and overall score.

Study	1	2	3	4	5	6	7	8	9	10	11	12	13	14	15	16	Total (n=16), n (%)
Lei et al [42], 2023	✓	✓			✓	✓	✓	✓			✓		✓		✓	✓	10 (62)
Czyz et al [43], 2023		✓			✓	✓	✓	✓	✓						✓	✓	8 (50)
Horwitz et al [44], 2023				✓	✓	✓	✓	✓	✓		✓				✓		8 (50)
Choo et al [45], 2022	✓	✓			✓	✓	✓	✓		✓	✓				✓		9 (56)
Cobo et al [46], 2021	✓	✓		✓			✓	✓			✓				✓	✓	8 (50)
Czyz et al [47], 2023	✓	✓		✓	✓	✓	✓	✓			✓	✓	✓	✓	✓	✓	13 (81)
Kaurin et al [48], 2022				✓	✓	✓	✓	✓			✓	✓	✓		✓	✓	10 (62)
Bonilla-Escribano et al [49], 2023	✓	✓		✓	✓	✓	✓	✓	✓		✓		✓		✓	✓	12 (75)
Marti-Puig et al [50], 2022		✓		✓	✓	✓	✓	✓	✓		✓				✓	✓	10 (62)
Peis et al [51], 2019		✓		✓	✓		✓								✓	✓	6 (38)
Choo et al [52], 2019	✓	✓			✓										✓		4 (25)
Wang et al [53], 2021		✓		✓	✓	✓	✓	✓	✓	✓	✓		✓	✓	✓	✓	13 (81)

### Data Synthesis

#### Overview

All (12/12, 100%) included studies were published since 2019, with 42% (5/12) of the studies published in 2023 [42-44,47,49], 25% (3/12) in 2022 [45,48,50], 17% (2/12) in 2021 [46,53], and 17% (2/12) in 2019 [51,52]. Studies were conducted in the United States [43,44,48,52], Spain [46,50], China [42], and in both France and Spain [49]. Suicidal ideation [42-46,48,49] was the suicide outcome that was most reported on across studies, with some (4/12, 33%) studies also reporting on suicide attempts [42,45] and self-harm [42,50]. Suicide outcomes were assessed using standardized measures, such as the Columbia Suicide Severity Rating Scale [43,47-49], the Patient Health Questionnaire-9 [42,44], and the Salzburg Suicide Process Questionnaire [49]. Some (2/12,17%) studies assessed suicide outcome using a single item on a standardized measure [42,44]; others (4/12, 33%) reported on suicide severity using scores from a standardized measure of suicidality [37,43,48,49]. In addition to suicide outcomes, studies also reported on the following related mental health outcomes: depression [44], sleep [46,47], stressful life events [52], drug and alcohol consumption [47,50], burdensomeness, hopelessness and connectedness [47], perceived social support [49], and conflict with others [50].

#### Settings

Studies were predominantly conducted in clinical settings. Settings included psychiatric hospitals [43,45,51-53], emergency departments [47,49], psychiatric outpatient clinics [43,48,49], medical residency programs [44], university mental health clinics [50], and a community sample recruited online [42], with some studies conducted across outpatient psychiatric and emergency departments [49]. In terms of study country, 6 (50%) of the 12 studies were conducted in the United States [43-45,47,48,52,53], 3 (25%) in Spain [46,50,51], 1 (8%) across France and Spain [49], and 1 (8%) study was conducted using an online recruitment of a community sample in China [42].

#### Participant Characteristics

The participant samples were predominantly clinical. Most (7/12, 58%) studies described participants as patients with psychiatric disorders [43,45,46,51,53], with some (3/12, 25%) targeting established vulnerable groups. In total, 33% (4/12) of the studies reported on patients with a diagnosis of BPD [45,48,50,52], 17% (2/12) described emergency department patients who had presented in a suicide crisis [47,49], 8% (1/12) reported on university students attending a mental health clinic [50], 8% (1/12) reported on first-year medical interns [44], and 8% (1/12) reported on a community sample recruited online who self-identified as sexual and gender minority individuals [42]. In terms of age profile, most (7/12, 58%) of the studies reported on adult samples of patients with psychiatric disorders [45,46,48,49,51-53], whereas 8% (1/12) of the studies reported on an adolescent sample aged between 13 and 17 years [43].

A combined total of 4398 participants were reported on across the 12 studies, with participant numbers ranging from 64 to 2479 and a mean participant number of 366.5. Participant gender, where this was reported, indicated a higher number of cisgender women [42,46].

#### Study Characteristics

Most (7/12, 58%) studies were observational cohort studies [42-44,46,47,49]—quantitative in nature—that applied ML to EMA data in order to predict suicide outcomes. Moreover, 8% (1/12) of the studies used ML to predict differences in EMA suicidal ideation after a randomized intervention [52]. Studies varied between prospective (7/12, 58%) and retrospective (5/12, 42%) designs. Most (7/12, 58%) studies collected data on frequent inpatient assessment of suicidal ideation and other outcomes to predict short-term suicide outcomes (predominantly suicidal ideation) after discharge. In total, 8% (1/12) of the studies [50] compared groups, including a subclinical, clinical, and healthy control group. Researchers applied AI to frequent inpatient assessment data (EMA) to predict short-term suicidal ideation after discharge [53] and at 1-month follow-up [42].

All (4/12, 33%) included retrospective studies used clinical file data [48,49,51,52], and 8% (1/12) used existing electronic health record (EHR) data [51], together with EMA data to predict defined outcomes that had occurred before the reported study commenced. In such cases, researchers reported on the extent to which the addition of EMA data boosted the predictive ability of the ML models applied.

Adherence to EMA protocol was reported in 4 (33%) of the 12 studies [42,43,49,53] and ranged from 52% [53] to 94.5% [42]. However, adherence to EMA protocol was defined differently across studies, and some (n=2, 17%) studies did not include the data of participants where the level of compliance fell below a specific threshold [48].

#### EMA Data Collection Procedures

Studies described collecting EMA data using smartphones [43,46-51,53] and wearable devices, such as Fitbits [44,47]. In most (7/12, 58%) of the studies, EMA data were collected daily [44,46-49] and at fixed time intervals [42,44,45,50]. Some (3/12, 25%) studies used daily EMA data collection at random times within the following parameters: between 10 AM and 10 PM [46], 4 times daily between 9:30 AM and 9:30 PM [43], or 6 times per day within a 12-hour period [48]. Peis et al [51] used an observational approach without fixed response procedures, whereby participants had to have interacted with the EMA component on at least 3 occasions to be eligible for inclusion in the analyses.

Most (9/12, 75%) studies collected EMA data during a single wave [43-45], some (2/12, 17%) collected data across 2 waves or before and after the intervention [52,53], and 8% (1/12) described 3 waves with daily EMA surveys by messages at 9:50 AM and 9:00 PM for 25 days [42]. The duration of EMA data collection periods reported ranged from 1 week to 130 days. All but one (11/12, 91%) study reported a fixed time period for all participants. Wang et al [53] reported that participants completed an EMA protocol for the duration of their hospital stay (mean 6.9, SD 5.4 days; range 2-46 days). Response latency or duration from prompt to response was largely unreported. Lei et al [42] and Kaurin et al [48] described participants having 2 hours and 1 hour to respond to EMA surveys, respectively.

#### Device Used

Most studies (8/12, 67%) described using smartphones [43,46-51,53], 17% (2/12) reported using Fitbit wearable devices [44,47], and 8% (1/12) used both [47]. In 17% (2/12) of the studies, the specific device used was not reported [42,45]. In terms of mobile apps and features used, 25% (3/12) of the studies reported using the MeMind app to record EMA data [46,49,51], 8% (1/12) used mobile text prompts [43], and 8% (1/12) used the Sinjur mobile app [50].

#### AI Applied and Results Reported

Studies used classification and regression trees (CARTs) [43,50], RF algorithms [44,49], elastic net models [44,53], and recurrent neural networks (RNNs) [45,51]. An overview of AI strategies applied in each study and a summary of results reported are presented in Table 3. Studies reported on mean cross-validation area under the curve (AUC) [42,44,47,49], mean sensitivity [43,46,47], mean specificity [42-44,49], mean positive predictive values (PPVs) [42,43,50], and mean negative predictive values [50]. Some (2/12, 17%) studies reported on root mean squared error (RMSE) [45,52]. The mean AUCs reported ranged from 0.71 [53] to 0.86 [43], mean sensitivity ranged from 0.64 [50] to 0.81 [43], and mean specificity ranged from 0.73 [44] to 0.86 [50]. A mean AUC of 0.89 was reported by Wang et al [53]. In this study, compared with a model using baseline data and one using mean-level real-time suicidal thoughts during hospitalization, predictive accuracy was best for the model using dynamic changes in real-time suicidal thoughts during hospitalization (AUC=0.89; IQR 0.81-0.97); this pattern of results held for other classification metrics (eg, accuracy, PPV, and Brier score) and when using different cross-validation procedures. Features assessing rapid fluctuations in suicidal thinking emerged as the strongest predictors of posthospital suicide attempts.

**Table 3:** Artificial intelligence (AI) strategies applied and results reported, with description of the type of AI applied to ecological momentary assessment (EMA) data in each study and the corresponding results reported.

Study	AI strategy applied	Results reported
Lei et al [42], 2023	Light Gradient Boosting Machine: the light boosting machine algorithm based on the GBDT^a^ algorithm	AUC^b^ (mean cross-validation) 0.8, 95% CI 0.78 to 0.81 Mean sensitivity 0.77, 95% CI 0.75 to 0.78 Mean specificity 0.78, 95% CI 0.76 to 0.79 Mean PPV^c^ 0.74, 95% CI 0.72 to 0.77
Czyz et al [43], 2023	Multilevel CART^d^	AUC (mean cross-validation) 0.86, SE 0.002 Mean sensitivity 0.81, SE 0.005 Mean specificity 0.82, SE 0.006 Mean PPV 0.74
Horwitz et al [44], 2023	Linear (ENR^e^) and nonlinear (RF^f^) algorithms	ENR: AUC (mean cross-validation) 0.736, SE 0.025 ENR: mean sensitivity 0.701 ENR: mean specificity 0.727 RF: AUC (mean cross-validation) 0.663, SE 0.016 RF: mean sensitivity 0.713 RF: mean specificity 0.699
Choo et al [45], 2022	RNNs^g^	RNNs based on baseline traits and momentary life events (RMSE^h^=3.61) Refitting RNNs without stressful life events (RMSE difference SD 0.92)
Cobo et al [46], 2021	Indian buffet process, a nonparametric Bayesian method	Four profiles identified Four suicide features accounted for >99.5% of the participant responses to the suicide ideation survey
Czyz et al [47], 2023	Mixed effects CARTs	EMA data: AUC (mean cross-validation) 0.84, SE 0.02 Sensor data: AUC (mean cross-validation) 0.56, SE 0.02
Kaurin et al [48], 2022	MSEM^i^	Model set 1: effects of others’ perceived warmth on SI^j^ No significant fixed effect at the within-person level (c=0.187, CI –0.017 to 0.404); significant fixed effect at the between-person level (βSI.W=–0.184, CI –0.357 to 0.000)
Bonilla-Escribano et al [49], 2023	A GMM^k^ RF algorithm	AUC (mean cross-validation) 0.74, 95% CI 0.68 to 0.78
Marti-Puig et al [50], 2022	CART LOSO^l^ cross-validation	AUC=0.847 Sensitivity=0.646 Specificity=0.855 PPV=0.145 NPV=0.985
Peis et al [51], 2019	RNNs	Model with attention and L=1 at the patient sequence achieved a recall close to 67% Recall=67.68 (SD –3.50) Accuracy=88.96 (SD –5.04) Precision=58.81 (SD –20.06) AUC=83.29 (SD –5.13)
Choo et al [52], 2019	Compared MEMs^m^ with RNNs	RNN: RMSE=5.32 MEM: RMSE=5.13
Wang et al [53], 2021	Elastic net models	AUC=0.71, IQR 0.55-0.88 for baseline data AUC=0.81, IQR 0.67-0.91 for real-time SI in hospital AUC=0.89, IQR 0.81-0.97 for dynamic changes in real-time SI After incorporating percentage missingness: AUC=0.93, IQR 0.90-1.00 for real-time SI in hospital and AUC=0.93; IQR 0.88-1.00 for dynamic changes in real-time SI

^a^GBDT: gradient boosting decision tree.

^b^AUC: area under the curve.

^c^PPV: positive predictive value.

^d^CART: classification and regression tree.

^e^ENR: elastic net regression.

^f^RF: random forest.

^g^RNN: recurrent neural network.

^h^RMSE: root mean squared error.

^i^MSEM: multiple structural equation modeling.

^j^SI: suicidal ideation.

^k^GMM: Gaussian mixture model.

^l^LOSO: leave one subject out.

^m^MEM: mixed effects regression models.

Studies that used RNNs reported RMSE=3.61 [47] to RMSE=5.13 [51]. Cobo et al [46] used the Indian buffet process, a nonparametric Bayesian method, to identify 4 profiles. Profile 1 was characterized by low values (ie, low probability of scoring positive) across all 32 suicide risk factors. Profiles 2 and 4 were characterized by a high desire for death, lack of wish to live, decreased appetite and tastelessness of food, and sleep problems; profile 4 also showed high values for negative emotions. Profile 3 was characterized by a lower desire for death and lower appetite and sleep symptoms, with high values of negative emotions. Researchers identified 4 suicide risk features that accounted for >99.5% of the participants’ responses. Peis et al [51] used deep sequential models to predict suicidal ideation from EHR and EMA data. The addition of EMA records boosted the system recall to predict suicidal ideation diagnosis from 48.13%, obtained exclusively from EHR-based methods, to 67.78%.

A small number of studies (2/12, 17%) compared models using self-report data and sensor data. Horowitz et al [44] used linear (elastic net regression [ENR]) and nonlinear (RF) ML algorithms to predict suicidal ideation at the first-quarter follow-up assessment, using 2 sets of variables (daily mood features only and daily mood features+passive-sensing features). For suicidal ideation, the ENR model using only mood variables over 92 days provided better predictive accuracy for suicidal ideation (AUC=0.736) relative to the model incorporating passive sensing (AUC=0.699). Acceptable accuracy (AUC>0.70) in the mood-only ENR model was maintained by week 7 but did not consistently meet this threshold when sensor data were included. Similarly, Peis et al [51] used deep sequential models and reported that the best-performing model incorporated features from EMAs and showed good predictive accuracy (mean cross-validated area under the receiver operating characteristic curve 0.84, SE 0.02), whereas the model that incorporated features from sensor data alone showed poor prediction (mean cross-validated AUC 0.56, SE 0.02).

In summary, of the 12 studies included in the review, most (n=8, 67%) were conducted in clinical settings with adults and reported on the application of ML to EMA data to predict near-term suicidal ideation. A high level of heterogeneity was noted in the reporting of EMA data collection procedures. Studies reported mean AUCs (0.74-0.86), sensitivity (0.64-0.81), specificity (0.73-0.86), and PPVs (0.72-0.77).

## Discussion

### Principal Findings

The application of AI to EMA data in suicide research is an emerging and rapidly growing area of research. To our knowledge, this is the first systematic review to synthesize findings on the application of AI to EMA data in the study of suicidal ideation and behaviors. The aim of this review was to synthesize published research on the application of AI strategies to EMA data in the study of suicide to identify the methodologies used, AI strategies applied, and results reported and to provide an adapted CREMAS framework for reporting AI applied to EMA data in mental health research. Of the 12 studies included in this review, most (n=8, 67%) investigated suicidal ideation among clinical populations, and high heterogeneity was found with respect to the reporting of study procedures, particularly EMA data collection procedures. When quality was appraised across the 16 recommended items put forward by the CREMAS of reporting standards, studies met between 4 and 13 of the recommended 16 items, with an average of 7 items met across studies. Studies performed poorly in their reporting of item 3, “description of EMA training procedures for participants,” and item 14, “treatment of missing data.” These results are consistent with the findings reported by Davanzo et al [30], who investigated EMA in BPD specifically as part of a systematic review and meta-analysis.

Reflective of the broad use of EMA data in suicide research, standardized reporting procedures could allow for more direct comparisons and benefit the overall advancement of the field. The development of a refined reporting framework (Multimedia Appendix 5) by the research team aims to support transparency and consistency in this area going forward. CREMAS was designed to facilitate greater standardization of reporting in EMA studies broadly and was initially applied in diet and activity research [41]. The framework designed following this review expands on this by including items specific to the reporting of AI procedures and results (items 21 and 22) and reporting of mental health outcomes specifically (items 4 and 5). In addition, inconsistencies in reporting, highlighted in previous reviews of both EMA and AI, informed the addition of more detailed items to better capture EMA data collection procedures (item 7). One item was added to capture the diversity of data sources in such research, that is, clinical files, sensor data, self-reports, etc. Finally, inconsistency in reporting across studies in this review informed the addition of specific items, for example, in relation to how AI results are reported, to facilitate future reviewers in synthesizing data [21,22].

In terms of AI strategies used, studies used CART [43,50], RF algorithms [44,49], elastic net models [44,46-49,51-53], and RNNs [45,51]. The best-performing models across accuracy, sensitivity, specificity, and PPV were multilevel CART [50] and elastic net models [53]. Previous research by Tang et al [54] investigating the effectiveness of explainable AI in suicide risk assessment indicated that DT, RF, and extreme gradient boosting models achieved the best results, while DT had the best performance with an AUC of 0.95 in non-EMA suicide datasets. In the application of AI to EMA data to predict suicidal ideation in the short term, studies reported mean AUCs (0.736-0.86), sensitivity (0.64-0.81), specificity (0.73-0.86), and PPVs (0.72-0.77). Studies that compared the accuracy of models found that the best-performing models required self-reported information derived from EMAs, whereas sensor-based data added negligible predictive accuracy. However, study authors noted that sensor-based assessments for sleep activity and heart rate were measured with emerging wearable sensor technology that may introduce more error than standard laboratory assessment [49].

Another notable finding is the importance of variability in prediction. In patients who were acutely suicidal, variability in suicidal thinking provided more information about suicide risk than average levels of suicidal thinking [26,53]. Wang et al [53] found that collecting real-time data about suicidal thinking in the course of a hospital stay significantly improved short-term prediction of posthospitalization suicide attempts. Models that included dynamic changes in suicidal thinking over time yielded the best prediction; features that captured rapid changes in suicidal thoughts were particularly strong predictors. Similarly, Bonilla-Escribano et al [49] reported that patients who were suicidal were best clustered into 2 groups with EMA data: low and high variability. The high-variability group showed more instability in all dimensions, particularly in social withdrawal, sleep measures, wish to live, and social support. Both clusters were separated by 10 clinical features (AUC=0.74), including depressive symptoms, cognitive instability, the intensity and frequency of passive suicidal ideation, and the occurrence of clinical events, such as suicide attempts or emergency visits during follow-up.

### Comparison With Prior Work

Findings are consistent with previous research, indicating that suicidal ideation severity varies considerably from hour to hour [55]. Survey noncompletion also emerged as an important predictor of posthospitalization suicide attempts [53]. Clinical initiatives to follow up with patients who are suicidal with ecological measures should take into account the existence of this high variability cluster and survey noncompletion to inform targeted support.

Researchers also examined the extent to which the EMA data type added to the predictive capabilities of a model. Peis et al [51] found that the addition of EMA records boosted the system recall to predict a suicidal ideation diagnosis from 48.13% obtained exclusively from EHR-based methods to 67.78%. Czyz et al [47] reported that the best-performing models in their study required self-reported information derived from EMAs, whereas sensor-based data had negligible predictive accuracy. The findings reported may also be reflective of the specific variables being measured using sensors. For example, previous research by Liu et al [56] achieved classification accuracy ranging from 96% to 98% for 3 risk levels across different modeling methods by applying ML to eye and head signal data. They identified that high-risk individuals experience psychomotor retardation and symptoms of anxiety and depression characterized by eye contact avoidance, slower blinks, and a downward eye gaze.

A common criticism of ML applied to EHR datasets retrospectively in health care research is that it is not known how models would perform if tested prospectively on truly independent patient samples. For example, in a non-EMA study [57], ML models predicted treatment outcomes among patients with schizophrenia with high accuracy within the trial in which the model was developed but performed no better than chance when applied out of sample. Researchers point to the challenge inherent in applying a model developed specifically in relation to one dataset to another clinical sample or setting. Salganik et al [58] measured the predictability of life outcomes broadly in a scientific mass collaboration. Despite using a rich dataset and applying ML methods optimized for prediction, the best predictions were not very accurate and only slightly better than those from a simple benchmark model. One important advantage of this review is that it reports on EMA data from predominantly prospective studies carried out in clinical settings with models identified a priori. EMA data collection procedures themselves may help to address challenges with EHR and other clinic-based data by reducing recall bias and capturing high levels of variability at an individual level.

### Limitations

While this is a growing area of research, a small number of published studies were identified and included in this review. Therefore, results should be interpreted with appropriate caution. Heterogeneity across data collection procedures, data analysis, and reporting of results hampered comparison. The authors argue that this may be understood in the context of a lack of standardized reporting guidelines, which motivated this work. AI and suicide research can also be dispersed across disciplines, reflecting diverse emphases revealing areas of expertise and research focus. Graham et al [59] argued that a diverse community of experts vested in mental health research and care, including scientists, clinicians, regulators, and patients, must communicate and collaborate to realize the full potential of AI. Although not an explicit aim of this review, the diversity of data collection procedures reported and the limited quantitative data available in general meant that it was not possible to compare groups. Further published research in this area would facilitate the completion of a meta-analysis to synthesize data on AI applied to EMA in predicting suicide outcomes across accuracy, sensitivity, specificity, and PPV and negative predictive value.

In terms of outcomes, most (7/12, 70%) studies reported on the ability of AI applied to EMA data to predict suicidal ideation rather than behavior. In addition, heterogeneity was noted in both the measurement and administration of suicidal ideation assessment measures, with some (2/12, 17%) studies relying on a single item of suicidal ideation for assessment [42,44]. There is a need for researchers to use established standardized guidelines when reporting model performance, such as the guidelines for developing and reporting ML predictive models in biomedical research [60].

Sample characteristics also limit the generalization of results reported outside of the specific clinical groups and settings in which studies were conducted. Authors reported that most participants were women [43,46], in some cases over 85% [46], and predominantly White [46,53]. Studies rarely included a control group, and the homogenous samples used (eg, patients with BPD, gender and sexual minority individuals, and patients who are admitted to the hospital for psychiatric care) while providing rich data regarding vulnerable groups limit generalization across clinical samples and settings.

### Future Research

Efforts to overcome heterogeneity in data collection and reporting procedures are needed to advance research in this area. The recent prioritization of open science and archival data sharing, together with greater integration of EHR in health systems and the proliferation of devices that facilitate EMA data collection, provides greater opportunities to apply innovative ML strategies to large mental health datasets. However, standardization of procedures and reporting is required to leverage such datasets to inform our understanding of suicide processes and develop targeted supports. The development of an adapted framework by the research team for reporting on studies applying AI strategies to EMA data in mental health research (Multimedia Appendix 5) aims to address this.

Future research should aim to validate clinical prediction models in different clinical samples. Chekroud et al [57] noted that this generally results in predictive performance measures that are lower but allows a more realistic indication of the potential of the model to inform clinical practice [61]. For AI, and in particular ML, to enhance clinical practice, the models developed must robustly predict outcomes for unseen, future patients. The generalizability of ML models beyond the data that were used to develop them is crucial for the clinical success of model efforts. In many cases, ML models have been evaluated in small samples of highly selected participants, and little is known about their potential for predicting mental health in other cohorts and settings [61]. Consequently, researchers have begun to depart from traditional participant pools toward more population-reflective, crowdsourced data collection [62]. Such big data approaches, recruiting participants worldwide and from diverse demographic backgrounds, may offer more representative data. Another key advantage of mobile assessment platforms is that they are more amenable to repeated and triggered assessments [61]. Further prospective EMA studies provide an opportunity to overcome many of the challenges inherent in generalizing models developed from EHR, clinical trial, or cross-sectional datasets.

Although EMA-based sampling strategies seem uniquely suited to capturing meaningful variation in suicidal thoughts and behaviors [63], there is uncertainty regarding how best to balance intensity and duration of assessment to capture this variation. Episodes of suicidal ideation have been found to be brief, with participants reporting most episodes to be shorter than an hour [64]. If the process assessed is faster than the assessment interval, EMA data and associations derived may not be inherently more valuable, particularly when measurements are not evenly spaced in time (eg, when EMA assessments are randomized throughout the day). The choice of an assessment and data analytic approach therefore requires theoretical justification and should be based on estimates of how long episodes of suicidal thinking may last. Future research in this area should systematically examine theoretically informed real-time sampling and modeling strategies of suicidal ideation.

RNNs, as found in this review [45,51,52], are potentially important tools for establishing model-free predictor importance. RNNs and other “black box” models have shown good prediction accuracy, but it can be difficult to explain the prediction made. A better understanding of how models operate can enable the detection of bias and faults of the model that can arise through biased training sets. Predictions from unexplainable models also pose substantial challenges to implementation, as the uptake of model predictors depends strongly on clinicians understanding and trusting them [62]. Evaluation of the importance of individual predictors in the context of highly correlated data presents challenges requiring appropriate methodology. Explainable predictive models, such as the Shapley Additive Explanatory approach, may be helpful in predicting and understanding the importance of features for suicide outcomes. Further research has shown that ML models with the Shapley Additive Explanatory approach are able to interpret and understand the nature of an individual’s predictions of suicidal behavior [65]. Future research using explainable predictive models may offer the opportunity to harness the predictive ability of ML models while providing transparency to predictions made. Clinically, such developments could inform personalized assessment and risk formulation and guide the development of clinical decision support tools.

### Conclusions

The findings of this review indicate that the application of AI to EMA data within suicide research is a small but burgeoning area. Substantial differences were apparent in reporting standards across studies, particularly with respect to EMA data collection procedures. Results indicate the strength of ML applied to self-report EMA data in the prediction of near-term suicidal ideation. The development of an adapted CREMAS reporting framework (Multimedia Appendix 6) by the research team aims to standardize reporting on the application of AI to EMA data in mental health research going forward.

An important goal of research in this area is to (1) identify models that will help predict who will experience suicide outcomes (necessary to inform clinical decision support tools) and (2) identify what supports work for whom and in what time frame (necessary to inform EMIs or just-in-time adaptive interventions). To achieve these goals, further prospective EMA studies applying ML models to diverse clinical samples, using standardized data collection and reporting procedures, are recommended. The application of AI to EMA data within suicide research offers the opportunity to move beyond a focus on *risk classification* toward dynamic data-driven *risk formulation*, consistent with recent clinical practice guidance [65]. In addition, integrating explainable AI could offer greater transparency to better inform clinician assessment and formulation and ultimately guide data-driven, personalized, and effective supports.

## Data Availability

The data included are made available through the published article and multimedia appendices. Further data regarding the quality appraisal process may be made available to researchers upon reasonable request to the study authors. The findings of this review are provided in the main text, tables, figures, and supplementary material of this paper. All studies included in this review are cited in the manuscript with full references available in the reference section.

## Appendix

Multimedia Appendix 1 Changes from preregistration.

Multimedia Appendix 2 Search strategy.

Multimedia Appendix 3 CREMAS (adapted STROBE [Strengthening the Reporting of Observational Studies in Epidemiology] Checklist for Reporting Ecological Momentary Assessment Studies).

Multimedia Appendix 4 PRISMA (Preferred Reporting Items for Systematic Reviews and Meta-Analyses) checklist.

Multimedia Appendix 5 Reporting framework.

Multimedia Appendix 6 Adapted framework applied to the studies included in this review.

## Abbreviations

AI: artificial intelligence
AUC: area under the curve
BPD: borderline personality disorder
CART: classification and regression tree
CREMAS: adapted STROBE Checklist for Reporting Ecological Momentary Assessment Studies
DT: decision tree
EHR: electronic health record
EMA: ecological momentary assessment
EMI: ecological momentary intervention
ENR: elastic net regression
ML: machine learning
PPV: positive predictive value
PRISMA: Preferred Reporting Items for Systematic Reviews and Meta-Analyses
RF: random forest
RMSE: root mean squared error
RNN: recurrent neural network
STROBE: Strengthening the Reporting of Observational Studies in Epidemiology

## References

[1] Suicide worldwide in 2019: global health estimates. World Health Organization.

[2] Franklin JC, Ribeiro JD, Fox KR, Bentley KH, Kleiman EM, Huang X, Musacchio KM, Jaroszewski AC, Chang BP, Nock MK (2017). Risk factors for suicidal thoughts and behaviors: a meta-analysis of 50 years of research. Psychol Bull.

[3] Joiner T (2005). Why People Die by Suicide.

[4] Rudd MD, Ellis TE (2006). Fluid vulnerability theory: a cognitive approach to understanding the process of acute and chronic suicide risk. Cognition and Suicide: Theory, Research, and Therapy.

[5] Klonsky ED, Pachkowski MC, Shahnaz A, May AM (2021). The three-step theory of suicide: description, evidence, and some useful points of clarification. Prev Med.

[6] O'Connor RC, Kirtley OJ (2018). The integrated motivational-volitional model of suicidal behaviour. Philos Trans R Soc Lond B Biol Sci.

[7] Shiffman S, Stone AA, Hufford MR (2008). Ecological momentary assessment. Annu Rev Clin Psychol.

[8] Stone AA, Shiffman S (1994). Ecological momentary assessment in behavioral medicine. Ann Behav Med.

[9] O'Connor RC, Nock MK (2014). The psychology of suicidal behaviour. Lancet Psychiatry.

[10] Nock MK, Borges G, Bromet EJ, Cha CB, Kessler RC, Lee S (2008). Suicide and suicidal behavior. Epidemiol Rev.

[11] Moskowitz DS, Young SN (2006). Ecological momentary assessment: what it is and why it is a method of the future in clinical psychopharmacology. J Psychiatry Neurosci.

[12] Jiménez-Muñoz L, Peñuelas-Calvo I, Díaz-Oliván I, Gutiérrez-Rojas L, Baca-García E, Porras-Segovia A (2022). Suicide prevention in your pocket: a systematic review of ecological momentary interventions for the management of suicidal thoughts and behaviors. Harv Rev Psychiatry.

[13] Rogers ML (2021). Feasibility and acceptability of ecological momentary assessment in a fully online study of community-based adults at high risk for suicide. Psychol Assess.

[14] Kivelä Liia, van der Does WA, Riese H, Antypa N (2022). Don't miss the moment: a systematic review of ecological momentary assessment in suicide research. Front Digit Health.

[15] Gee BL, Han J, Benassi H, Batterham PJ (2020). Suicidal thoughts, suicidal behaviours and self-harm in daily life: a systematic review of ecological momentary assessment studies. Digit Health.

[16] Torous J, Kiang MV, Lorme J, Onnela JP (2016). New tools for new research in psychiatry: a scalable and customizable platform to empower data driven smartphone research. JMIR Ment Health.

[17] Ferreri F, Bourla A, Mouchabac S, Karila L (2018). e-addictology: an overview of new technologies for assessing and intervening in addictive behaviors. Front Psychiatry.

[18] Bourla A, Mouchabac S, El Hage W, Ferreri F (2018). e-PTSD: an overview on how new technologies can improve prediction and assessment of posttraumatic stress disorder (PTSD). Eur J Psychotraumatol.

[19] Sequeira L, Battaglia M, Perrotta S, Merikangas KR, Strauss J (2019). Digital phenotyping with mobile and wearable devices: advanced symptom measurement in child and adolescent depression. J Am Acad Child Adolesc Psychiatry.

[20] Bernert RA, Hilberg AM, Melia R, Kim JP, Shah NH, Abnousi F (2020). Artificial intelligence and suicide prevention: a systematic review of machine learning investigations. Int J Environ Res Public Health.

[21] Lejeune A, Le Glaz A, Perron PA, Sebti J, Baca-Garcia E, Walter M, Lemey C, Berrouiguet S (2022). Artificial intelligence and suicide prevention: a systematic review. Eur Psychiatry.

[22] Burke TA, Jacobucci R, Ammerman BA, Piccirillo M, McCloskey MS, Heimberg RG, Alloy LB (2018). Identifying the relative importance of non-suicidal self-injury features in classifying suicidal ideation, plans, and behavior using exploratory data mining. Psychiatry Res.

[23] Fonseka TM, Bhat V, Kennedy SH (2019). The utility of artificial intelligence in suicide risk prediction and the management of suicidal behaviors. Aust N Z J Psychiatry.

[24] Kessler RC, Hwang I, Hoffmire CA, McCarthy JF, Petukhova MV, Rosellini AJ, Sampson NA, Schneider AL, Bradley PA, Katz IR, Thompson C, Bossarte RM (2017). Developing a practical suicide risk prediction model for targeting high-risk patients in the Veterans Health Administration. Int J Methods Psychiatr Res.

[25] Choo C, Diederich J, Song I, Ho R (2014). Cluster analysis reveals risk factors for repeated suicide attempts in a multi-ethnic Asian population. Asian J Psychiatr.

[26] Schafer KM, Kennedy G, Gallyer A, Resnik P (2021). A direct comparison of theory-driven and machine learning prediction of suicide: a meta-analysis. PLoS One.

[27] Morgiève M, Genty C, Azé J, Dubois J, Leboyer M, Vaiva G, Berrouiguet S, Courtet PA (2020). A digital companion, the Emma app, for ecological momentary assessment and prevention of suicide: quantitative case series study. JMIR Mhealth Uhealth.

[28] Melia R, Francis K, Duggan J, Bogue J, O'Sullivan M, Young K, Chambers D, McInerney SJ, O'Dea E, Bernert R (2023). Using a safety planning mobile app to address suicidality in young people attending community mental health services in Ireland: protocol for a pilot randomized controlled trial. JMIR Res Protoc.

[29] Parrish EM, Chalker SA, Cano M, Moore RC, Pinkham AE, Harvey PD, Joiner T, Lieberman A, Granholm E, Depp CA (2021). Ecological momentary assessment of interpersonal theory of suicide constructs in people experiencing psychotic symptoms. J Psychiatr Res.

[30] Davanzo A, D Huart D, Seker S, Moessner M, Zimmermann R, Schmeck K, Behn A (2023). Study features and response compliance in ecological momentary assessment research in borderline personality disorder: systematic review and meta-analysis. J Med Internet Res.

[31] Heron KE, Smyth JM (2010). Ecological momentary interventions: incorporating mobile technology into psychosocial and health behaviour treatments. Br J Health Psychol.

[32] Coppersmith DD, Dempsey W, Kleiman EM, Bentley KH, Murphy SA, Nock MK (2022). Just-in-time adaptive interventions for suicide prevention: promise, challenges, and future directions. Psychiatry.

[33] Nahum-Shani I, Smith SN, Spring BJ, Collins LM, Witkiewitz K, Tewari A, Murphy SA (2018). Just-in-time adaptive interventions (JITAIs) in mobile health: key components and design principles for ongoing health behavior support. Ann Behav Med.

[34] Riley W, Obermayer J, Jean-Mary J (2008). Internet and mobile phone text messaging intervention for college smokers. J Am Coll Health.

[35] Patrick K, Raab F, Adams MA, Dillon L, Zabinski M, Rock CL, Griswold WG, Norman GJ (2009). A text message-based intervention for weight loss: randomized controlled trial. J Med Internet Res.

[36] Bryan CJ, Wastler H, Allan N, Khazem LK, Rudd MD (2022). Just-in-time adaptive interventions (JITAIs) for suicide prevention: tempering expectations. Psychiatry.

[37] Page MJ, McKenzie JE, Bossuyt PM, Boutron I, Hoffmann TC, Mulrow CD, Shamseer L, Tetzlaff JM, Akl EA, Brennan SE, Chou R, Glanville J, Grimshaw JM, Hróbjartsson A, Lalu MM, Li T, Loder EW, Mayo-Wilson E, McDonald S, McGuinness LA, Stewart LA, Thomas J, Tricco AC, Welch VA, Whiting P, Moher D (2021). The PRISMA 2020 statement: an updated guideline for reporting systematic reviews. BMJ.

[38] Melia R, Wilson E The application of artificial intelligence to ecological momentary assessment data in suicide research: a systematic review. PROSPERO.

[39] Melia R, Schafer K, Wilson E, Rogers ML, Joiner TE (2024). The application of artificial intelligence strategies to ecological momentary assessment data in suicide research. J Med Internet Res (Forthcoming).

[40] Ouzzani M, Hammady H, Fedorowicz Z, Elmagarmid A (2016). Rayyan-a web and mobile app for systematic reviews. Syst Rev.

[41] Liao Y, Skelton K, Dunton G, Bruening M (2016). A systematic review of methods and procedures used in ecological momentary assessments of diet and physical activity research in youth: an adapted STROBE checklist for reporting EMA studies (CREMAS). J Med Internet Res.

[42] Lei C, Qu D, Liu K, Chen R (2023). Ecological momentary assessment and machine learning for predicting suicidal ideation among sexual and gender minority individuals. JAMA Netw Open.

[43] Czyz EK, Koo HJ, Al-Dajani N, King CA, Nahum-Shani I (2023). Predicting short-term suicidal thoughts in adolescents using machine learning: developing decision tools to identify daily level risk after hospitalization. Psychol Med.

[44] Horwitz AG, Kentopp SD, Cleary J, Ross K, Wu Z, Sen S, Czyz EK (2023). Using machine learning with intensive longitudinal data to predict depression and suicidal ideation among medical interns over time. Psychol Med.

[45] Choo T, Galfalvy HC, Stanley B (2022). P694. Effects of life events on suicidal ideation in EMA data using recurrent neural network prediction. Biological Psychiatry.

[46] Cobo A, Porras-Segovia A, Pérez-Rodríguez MM, Artés-Rodríguez A, Barrigón ML, Courtet P, Baca-García E (2021). Patients at high risk of suicide before and during a COVID-19 lockdown: ecological momentary assessment study. BJPsych Open.

[47] Czyz EK, King CA, Al-Dajani N, Zimmermann L, Hong V, Nahum-Shani I (2023). Ecological momentary assessments and passive sensing in the prediction of short-term suicidal ideation in young adults. JAMA Netw Open.

[48] Kaurin A, Dombrovski AY, Hallquist MN, Wright AG (2022). Momentary interpersonal processes of suicidal surges in borderline personality disorder. Psychol Med.

[49] Bonilla-Escribano P, Ramírez D, Baca-García E, Courtet P, Artés-Rodríguez A, López-Castromán J (2023). Multidimensional variability in ecological assessments predicts two clusters of suicidal patients. Sci Rep.

[50] Marti-Puig P, Capra C, Vega D, Llunas L, Solé-Casals J (2022). A machine learning approach for predicting non-suicidal self-injury in young adults. Sensors (Basel).

[51] Peis I, Olmos PM, Vera-Varela C, Barrigon ML, Courtet P, Baca-Garcia E, Artes-Rodriguez A (2019). Deep sequential models for suicidal ideation from multiple source data. IEEE J Biomed Health Inform.

[52] Choo TH, Galfalvy H, Stanley B (2019). F150. Machine learning method predicting differences in EMA suicidal ideation scores after randomized treatment. Biological Psychiatry.

[53] Wang SB, Coppersmith DD, Kleiman EM, Bentley KH, Millner AJ, Fortgang R, Mair P, Dempsey W, Huffman JC, Nock MK (2021). A pilot study using frequent inpatient assessments of suicidal thinking to predict short-term postdischarge suicidal behavior. JAMA Netw Open.

[54] Tang H, Miri Rekavandi A, Rooprai D, Dwivedi G, Sanfilippo FM, Boussaid F, Bennamoun M (2024). Analysis and evaluation of explainable artificial intelligence on suicide risk assessment. Sci Rep.

[55] Hallensleben N, Glaesmer H, Forkmann T, Rath D, Strauss M, Kersting A, Spangenberg L (2019). Predicting suicidal ideation by interpersonal variables, hopelessness and depression in real-time. An ecological momentary assessment study in psychiatric inpatients with depression. Eur Psychiatry.

[56] Liu S, Lu C, Alghaminem S, Gotoh L, Breazeal C, Park K (2022). Explainable artificial intelligence for suicide risk assessment using eye activities and hand gestures. Proceedings of the 2022 Artificial Intelligence in HCI: 3rd International Conference, AI-HCI 2022, Held as Part of the 24th HCI International Conference.

[57] Chekroud AM, Hawrilenko M, Loho H, Bondar J, Gueorguieva R, Hasan A, Kambeitz J, Corlett PR, Koutsouleris N, Krumholz HM, Krystal JH, Paulus M (2024). Illusory generalizability of clinical prediction models. Science.

[58] Salganik MJ, Lundberg I, Kindel AT, Ahearn CE, Al-Ghoneim K, Almaatouq A, Altschul DM, Brand JE, Carnegie NB, Compton RJ, Datta D, Davidson T, Filippova A, Gilroy C, Goode BJ, Jahani E, Kashyap R, Kirchner A, McKay S, Morgan AC, Pentland A, Polimis K, Raes L, Rigobon DE, Roberts CV, Stanescu DM, Suhara Y, Usmani A, Wang EH, Adem M, Alhajri A, AlShebli B, Amin R, Amos RB, Argyle LP, Baer-Bositis L, Büchi M, Chung B, Eggert W, Faletto G, Fan Z, Freese J, Gadgil T, Gagné J, Gao Y, Halpern-Manners A, Hashim SP, Hausen S, He G, Higuera K, Hogan B, Horwitz IM, Hummel LM, Jain N, Jin K, Jurgens D, Kaminski P, Karapetyan A, Kim EH, Leizman B, Liu N, Möser M, Mack AE, Mahajan M, Mandell N, Marahrens H, Mercado-Garcia D, Mocz V, Mueller-Gastell K, Musse A, Niu Q, Nowak W, Omidvar H, Or A, Ouyang K, Pinto KM, Porter E, Porter KE, Qian C, Rauf T, Sargsyan A, Schaffner T, Schnabel L, Schonfeld B, Sender B, Tang JD, Tsurkov E, van Loon A, Varol O, Wang X, Wang Z, Wang J, Wang F, Weissman S, Whitaker K, Wolters MK, Woon WL, Wu J, Wu C, Yang K, Yin J, Zhao B, Zhu C, Brooks-Gunn J, Engelhardt BE, Hardt M, Knox D, Levy K, Narayanan A, Stewart BM, Watts DJ, McLanahan S (2020). Measuring the predictability of life outcomes with a scientific mass collaboration. Proc Natl Acad Sci U S A.

[59] Graham S, Depp C, Lee EE, Nebeker C, Tu X, Kim H, Jeste DV (2019). Artificial intelligence for mental health and mental illnesses: an overview. Curr Psychiatry Rep.

[60] Luo W, Phung D, Tran T, Gupta S, Rana S, Karmakar C, Shilton A, Yearwood J, Dimitrova N, Ho TB, Venkatesh S, Berk M (2016). Guidelines for developing and reporting machine learning predictive models in biomedical research: a multidisciplinary view. J Med Internet Res.

[61] Riley RD, Debray TP, Collins GS, Archer L, Ensor J, van Smeden M, Snell KIE (2021). Minimum sample size for external validation of a clinical prediction model with a binary outcome. Stat Med.

[62] Hauser TU, Skvortsova V, De Choudhury M, Koutsouleris N (2022). The promise of a model-based psychiatry: building computational models of mental ill health. Lancet Digit Health.

[63] Gratch I, Choo T, Galfalvy H, Keilp JG, Itzhaky L, Mann JJ, Oquendo MA, Stanley B (2021). Detecting suicidal thoughts: the power of ecological momentary assessment. Depress Anxiety.

[64] Nock MK, Prinstein MJ, Sterba SK (2009). Revealing the form and function of self-injurious thoughts and behaviors: a real-time ecological assessment study among adolescents and young adults. J Abnorm Psychol.

[65] Nordin N, Zainol Z, Mohd Noor MH, Chan LF (2023). An explainable predictive model for suicide attempt risk using an ensemble learning and Shapley Additive Explanations (SHAP) approach. Asian J Psychiatr.

