# Supplementary material for: The Application of AI to Ecological Momentary Assessment Data in Suicide Research: Systematic Review
Source: J Med Internet Res. 2025 Apr 17;27:e63192. doi: 10.2196/63192 (PMC12046261; doi:10.2196/63192)
Supplement: Multimedia Appendix 1 [file jmir_v27i1e63192_app1.docx]

Pre-registration of Study Protocol PROSPERO: CRD42023440218

Open Science Framework: <https://doi.org/10.17605/OSF.IO/NZWUJ>

Changes from study protocol to manuscript submission:

1. The search strategy dates were extended to facilitate a final search of published research prior to submission to JMIR. Search dates are as follows: Initial search 20^th^ July 2023 (inception to July 2023), additional search 15^th^ August 2023 (inception to August 2023), final search June 2^nd^ 2024 (inception to June 2^nd^ 2024).
2. A meta-analysis was planned when first developing the study protocol, if data available in included studies would permit. This was not the case as a small number of studies were included in the review and studies reported on different data types and AI strategies that rendered a meta-analysis inappropriate. Therefore, a narrative synthesis of study findings is provided here.
