# Supplementary material for: The Application of AI to Ecological Momentary Assessment Data in Suicide Research: Systematic Review
Source: J Med Internet Res. 2025 Apr 17;27:e63192. doi: 10.2196/63192 (PMC12046261; doi:10.2196/63192)
Supplement: Multimedia Appendix 2 [file jmir_v27i1e63192_app2.docx]

Search Strategy used for EMBASE

This strategy was adapted for other databases used.

| Suicide | Artificial Intelligence | Ecological Momentary Assessment |
| --- | --- | --- |
| Suicid*/  Suicide ideation/  Suicidal/  Suicide attempt/  Suicide death/ | Artificial Intelligence/  AI/  Machine Learning /  ML | EMA/  Ecological Momentary Assessment/  Experience Sampling/  Ambulatory Assessment |

1. Suicide
2. Artificial Intelligence
3. Ecological Momentary Assessment

1 AND 2 AND 3
