# Supplementary material for: The Application of AI to Ecological Momentary Assessment Data in Suicide Research: Systematic Review
Source: J Med Internet Res. 2025 Apr 17;27:e63192. doi: 10.2196/63192 (PMC12046261; doi:10.2196/63192)
Supplement: Multimedia Appendix 3 [file jmir_v27i1e63192_app3.docx]

Adapted STROBE Checklist for Reporting EMA Studies (CREMAS)

| Title |  |  |
| --- | --- | --- |
| 1. Title | Include ecological momentary assessment in title and key words |  |
| Introduction |  |  |
| 2. Rationale | Briefly introduce the concept of EMA and provide reasons for utilizing EMA for this study or topic of interests |  |
| Methods |  |  |
| 3. Training | Indicate if, and by what methods, training of participants for EMA protocol was used |  |
| 4. Technology | Describe what technology, if any, was used. Include the following information: device (eg, mobile phone, portable computer), model (eg, Nexus 4, iPod), operating system (eg, Android, Windows), and EMA program name |  |
| 5. Wave Duration | State the number of waves for the study (eg, 2 monitoring periods over the course of 1 year) |  |
| 6. Monitoring period | State the number of days each wave of the study lasted, and how many weekdays versus weekend days |  |
| 7. Prompting design | Indicate the prompting strategy used for the study (eg, event-based, interval-based, or a combination of the two). If using interval-based strategy, indicate what type of schedule is used (eg, fixed, random, or hybrid interval) |  |
| 8. Prompt Frequency | Intended frequency of prompts per day. Break down by weekdays and weekend days if applicable |  |
| 9. Design features | Describe any design feature to address potential sources of bias (eg, reactivity) or participant burden (eg, EMA questions appearing in different orders) |  |
| Results |  |  |
| 10. Attrition | Indicate participant attrition throughout the study; report attrition rates both by monitoring days and waves, if applicable |  |
| 11. Prompt delivery | Report number of EMA prompts that were planned to be delivered. If possible, also report the number of EMA prompts that were actually received by participants and indicate reasons for why prompts were not sent out (eg, technical issues or participant noncompliance reason such as phone was powered off) |  |
| 12. Latency | Report the amount of time from prompt signal to answering of prompt |  |
| 13. Compliance rate | Report total answered EMA prompts across all subjects and the average number of EMA prompts answered per person. Report compliance rate both by monitoring days and waves, if applicable. Indicate reasons for noncompliance, if known |  |
| 14. Missing data | Report whether EMA compliance is related to demographic or time-varying variables |  |
| Discussion |  |  |
| 15. Limitations | Discuss limitations of the study, taking into account sources of potential bias when using EMA methods (eg, reactivity, use of technology) |  |
| 16. Conclusions | Provide a general interpretation of results and discuss the benefits of using EMA |  |
