# Supplementary material for: The Application of AI to Ecological Momentary Assessment Data in Suicide Research: Systematic Review
Source: J Med Internet Res. 2025 Apr 17;27:e63192. doi: 10.2196/63192 (PMC12046261; doi:10.2196/63192)
Supplement: Multimedia Appendix 5 [file jmir_v27i1e63192_app5.docx]

An adapted framework for reporting on AI applied to EMA data in mental health research

| **Topic** | **Item Number** | **Checklist Item** | **Page Number Reported** |
| --- | --- | --- | --- |
| Description | 1 | Include ecological momentary assessment (EMA) in the title and abstract |  |
|  | 2 | Include Artificial Intelligence (AI) / type of AI used in the title and abstract |  |
|  | 3 | Describe briefly both EMA and AI and their utility in this area of research |  |
| Outcomes | 4 | Identify mental health outcomes studied |  |
|  | 5 | Identify standardised measure(s) used to collect outcome data |  |
|  |  | Describe all sources of data included i.e. mobile-based EMA, electronic Health Records, clinical file |  |
| Technology | 6 | Identify device(s) used to collect EMA data |  |
|  |  | Identity EMA programme or application used to facilitate data collection |  |
| EMA Data Collection | 7 | Provide a description of the EMA data collection procedures used |  |
|  | 8 | Describe methods of training of participants for EMA data collection procedures used |  |
|  | 9 | State the number of waves for the study (e.g. 2 monitoring periods over the course of 1 year) |  |
|  | 10 | State the number of days each wave of the study lasted |  |
|  | 11 | Indicate the prompting strategy used i.e. event-based, interval-based. If using interval-based strategy, indicate what type of schedule used i.e. fixed, random, or hybrid interval |  |
|  | 12 | Describe latency period between EMA prompt and participant response |  |
|  | 13 | Report on compliance with EMA data collection procedures |  |
|  | 14 | Report on how missing data was treated |  |
|  | 15 | Identify intended frequency of prompts per day |  |
| Study Design | 16 | Identify and describe study type and design i.e. retrospective or prospective, RCT / case series / cohort control |  |
|  | 17 | Describe any design feature to address potential sources of bias (e.g., reactivity) or participant burden |  |
| Participants | 18 | Describe population studied and report on number of participants |  |
|  | 19 | Report on attrition from the study |  |
| Setting | 20 | Describe the setting(s) in which the study was conducted (country, clinical / community setting). If enrolment was conducted online, what setting was recruitment aimed at? |  |
| Artificial Intelligence | 21 | Identify the type of AI used |  |
|  | 22 | Provide description of the data analytic strategies used, the results of those analyses, and their parameters (for example, and where appropriate, report on mean Area Under the Curve, Specificity, Sensitivity, Positive Predictive Values) |  |
| Limitations | 23 | Discuss the limitations of the methodologies used (EMA data collection procedures, AI data analytics applied) |  |
| Conclusion | 24 | Provide clear conclusion or take-home message regarding the application of AI to EMA data in mental health research |  |
