# Supplementary material for: The Application of AI to Ecological Momentary Assessment Data in Suicide Research: Systematic Review
Source: J Med Internet Res. 2025 Apr 17;27:e63192. doi: 10.2196/63192 (PMC12046261; doi:10.2196/63192)
Supplement: Multimedia Appendix 6 [file jmir_v27i1e63192_app6.docx]

Adapted Framework Applied to Studies Included in the Review

| **Topic** | **Checklist Item** | **[42]** | **[43]** | **[44]** | **[45]** | **[46]** | **[47]** | **[48]** | **[49]** | **[50]** | **[51]** | **[52]** | **[53]** |
| --- | --- | --- | --- | --- | --- | --- | --- | --- | --- | --- | --- | --- | --- |
| Description | Include ecological momentary assessment (EMA) in the title and abstract | √ | X | X | √ | √ | X | √ | √ | X | X | √ | X |
|  | Include Artificial Intelligence (AI) / type of AI used in the title and abstract | √ | √ | √ | √ | X | √ | √ | X | X | X | X | √ |
|  | Describe briefly both EMA and AI and their utility in this area of research | √ | √ | √ | √ | √ | X | √ | X | X | X | √ | X |
| Outcomes | Identify mental health outcomes studied | √ | √ | √ | X | √ | √ | √ | √ | √ | √ | √ | √ |
|  | Identify standardised measure(s) used to collect outcome data | √ | √ | √ | X | √ | √ | √ | √ | √ | √ | √ | √ |
|  | Describe all sources of data included i.e. mobile-based EMA, electronic Health Records, clinical file | √ | √ | √ | √ | √ | √ | √ | √ | √ | √ | √ | √ |
| Technology | Identify device(s) used to collect EMA data | X | √ | √ | X | √ | √ | X | √ | √ | √ | √ | √ |
|  | Identity EMA programme or application used to facilitate data collection | X | X | √ | X | √ | X | X | √ | √ | √ | √ | √ |
| EMA Data Collection | Provide a description of the EMA data collection procedures used | √ | √ | √ | √ | √ | √ | X | √ | √ | √ | √ | √ |
|  | Describe methods of training of participants for EMA data collection procedures used | X | X | X | X | X | X | √ | X | X | √ | X | √ |
|  | State the number of waves for the study (e.g. 2 monitoring periods over the course of 1 year) | √ | √ | √ | √ | X | √ | X | √ | √ | √ | √ | √ |
|  | State the number of days each wave of the study lasted | √ | √ | √ | √ | X | X | X | √ | √ | √ | √ | √ |
|  | Indicate the prompting strategy used i.e. event-based, interval-based. If using interval-based strategy, indicate what type of schedule used i.e. fixed, random, or hybrid interval | √ | √ | √ | √ | √ | √ | X | √ | √ | √ | √ | √ |
|  | Describe latency period between EMA prompt and participant response | √ | X | X | X | X | X | √ | X | X | X | X | X |
|  | Report on compliance with EMA data collection procedures | √ | X | X | X | X | X | X | √ | √ | √ | √ | X |
|  | Report on how missing data was treated | √ | X | √ | X | X | X | X | X | X | X | X | X |
|  | Identify intended frequency of prompts per day | √ | √ | √ | X | √ | √ | X | √ | √ | √ | √ | √ |
| Study Design | Identify and describe study type and design i.e. retrospective or prospective, RCT / case series / cohort control | √ | √ | √ | √ | X | √ | √ | √ | √ | √ | √ | √ |
|  | Describe any design feature to address potential sources of bias (e.g., reactivity) or participant burden | √ | X | √ | X | X | X | X | X | X | √ | X | √ |
| Participants | Describe population studied and report on number of participants | √ | √ | √ | √ | √ | √ | √ | √ | √ | √ | √ | √ |
|  | Report on attrition from the study | X | X | X | X | X | X | X | X | X | X | X | X |
| Setting | Describe the setting(s) in which the study was conducted (country, clinical / community setting). If enrolment was conducted online, what setting was recruitment aimed at? | √ | √ | √ | √ | √ | √ | √ | √ | √ | √ | √ | √ |
| Artificial Intelligence | Identify the type of AI used | √ | √ | √ | √ | √ | √ | √ | √ | √ | √ | √ | √ |
|  | Provide description of the data analytic strategies used, the results of those analyses, and their parameters (for example, and where appropriate, report on mean Area Under the Curve, Specificity, Sensitivity, Positive Predictive Values) | √ | √ | √ | √ | √ | √ | √ | √ | √ | √ | √ | √ |
| Limitations | Discuss the limitations of the methodologies used (EMA data collection procedures, AI data analytics applied) | √ | √ | √ | √ | √ | √ | X | √ | √ | √ | √ | √ |
| Conclusion | Provide clear conclusion or take-home message regarding the application of AI to EMA data in mental health research | √ | √ | √ | √ | √ | √ | √ | √ | √ | √ | √ | √ |
| Total |  | 21 | 18 | 21 | 15 | 16 | 16 | 13 | 19 | 18 | 20 | 20 | 20 |

Reference List

42. Lei C, Qu D, Liu K, Chen R. Ecological Momentary Assessment and Machine Learning for Predicting Suicidal Ideation Among Sexual and Gender Minority Individuals, JAMA Network Open. September, 2023, 6(9):e2333164. doi: 10.1001/jamanetworkopen.2023.33164. PMID: 37695580; PMCID: PMC10495869.

43. Czyz EK, Koo HJ, Al-Dajani N, King CA, Nahum-Shani I. Predicting short-term suicidal thoughts in adolescents using machine learning: developing decision tools to identify daily level risk after hospitalization. Psychological Medicine. 2023, 53(7):2982-2991. doi:10.1017/S0033291721005006

44. Horwitz AG, Kentopp SD, Cleary J, Ross K, Wu Z, San S, Czyz E. Using machine learning with intensive longitudinal data to predict depression and suicidal ideation among medical interns over time. Psychological Medicine, 2023, 53(12):5778-5785. doi:10.1017/S0033291722003014

45. Choo T, Galfalvy HC, Stanley BH. P694. Effects of Life Events on Suicidal Ideation in EMA Data Using Recurrent Neural Network Prediction. Biological Psychiatry, 2022, 91, S371-S372.

46. Cobo A. Porras-Segovia A, Pérez-Rodríguez MM. et al. Patients at high risk of suicide before and during a COVID-19 lockdown: ecological momentary assessment study. BJPsych Open, 2021, 7(3): e82. doi:10.1192/bjo.2021.43

47. Czyz EK, King CA, Al-Dajani N, Zimmermann L, Hong V, Nahum-Shani I. Ecological Momentary Assessments and Passive Sensing in the Prediction of Short-Term Suicidal Ideation in Young Adults, JAMA Network Open. August, 2023, 1;6(8):e2328005. doi: 10.1001/jamanetworkopen.2023.28005. PMID: 37552477; PMCID: PMC10410485.

48. Kaurin A, Dombrovski AY, Hallquist MN, Wright AGC. Momentary interpersonal processes of suicidal surges in borderline personality disorder. Psychological Medicine, 2022, 52(13):2702-2712. doi:10.1017/S0033291720004791

49. Bonilla-Escribano P, Ramírez D, Baca-García E, Courtet P, Artés-Rodríguez A, López-Castromán J. Multidimensional variability in ecological assessments predicts two clusters of suicidal patients. Scientific Reports, 2023, 13(1):3546. doi: 10.1038/s41598-023-30085-1. PMID: 36864070; PMCID: PMC9981613.

50. Marti-Puig P, Capra C, Vega D, Llunas L, Solé-Casals J. A Machine Learning Approach for Predicting Non-Suicidal Self-Injury in Young Adults. Sensors (Basel). June, 2022, 24;22(13):4790. doi: 10.3390/s22134790. PMID: 35808286; PMCID: PMC9269418.

51. Peis I, Olmos PM, Vera-Varela C, Barrigan ML, Courtet P, Baca-Garcia G, Arte-Rodriguez A. Deep sequential models for suicidal ideation from multiple source data. IEEE J Biomed Health Inform, 2019, 223(6):2286-2293. doi:10.1109/JBHI.2019.2919270

52. Choo TH, Galfalvy H, Stanley B. Machine Learning Method Predicting Differences in EMA Suicidal Ideation Scores After Randomized Treatment. Biological Psychiatry. May, 2019; 85: S13-S379

53. Wang SB, Coppersmith DDL, Kleiman EM, Bentley KH, Millner AJ, Fortgang R, Mair P, Dempsey W, Huffman JC, Nock MK. A Pilot Study Using Frequent Inpatient Assessments of Suicidal Thinking to Predict Short-Term Postdischarge Suicidal Behavior. JAMA Network Open, 2021, 4(3):e210591. doi: 10.1001/jamanetworkopen.2021.0591. PMID: 33687442; PMCID: PMC7944382.
